# SMALL LEAF AND BUSHY1 controls organ size and lateral branching by modulating the stability of BIG SEEDS1 in *Medicago truncatula*

**DOI:** 10.1111/nph.16449

**Published:** 2020-02-19

**Authors:** Pengcheng Yin, Qingxia Ma, Hui Wang, Dan Feng, Xianbing Wang, Yanxi Pei, Jiangqi Wen, Million Tadege, Lifang Niu, Hao Lin

**Affiliations:** ^1^ Biotechnology Research Institute Chinese Academy of Agricultural Sciences Beijing 100081 China; ^2^ College of Biological Sciences China Agricultural University Beijing 100193 China; ^3^ College of Life Science Shanxi University Taiyuan 030006 China; ^4^ Department of Plant and Soil Sciences Institute for Agricultural Biosciences Oklahoma State University Ardmore OK 73401 USA; ^5^ Noble Research Institute, LLC Ardmore OK 73401 USA

**Keywords:** BIG SEEDS1, F‐box protein, lateral branching, *Medicago truncatula*, organ size, SCF complex, SMALL LEAF AND BUSHY1

## Abstract

- Organ size is a major agronomic trait that determines grain yield and biomass production in crops. However, the molecular mechanisms controlling organ size, especially in legumes, are poorly understood.
- Using forward genetic approaches in a *Tnt1* insertion mutant population of the model legume *Medicago truncatula*, we identified *SMALL LEAF AND BUSHY1* (*SLB1*), which is required for the control of organ size and lateral branching.
- Loss of function of *SLB1* led to reduced leaf and flower size but increased lateral branch formation in *M. truncatula*. *SLB1* encodes an F‐box protein, an orthologue of *Arabidopsis thaliana* STERILE APETALA (SAP), that forms part of an SKP1/Cullin/F‐box E3 ubiquitin ligase complex. Biochemical and genetic analyses revealed that SLB1 controls *M. truncatula* organ growth and lateral branching by modulating the stability of BIG SEEDS1 (BS1). Moreover, the overexpression of *SLB1* increased seed and leaf size in both *M. truncatula* and soybean (*Glycine max*), indicating functional conservation.
- Our findings revealed a novel mechanism by which SLB1 targets BS1 for degradation to regulate *M. truncatula* organ size and shoot branching, providing a new genetic tool for increasing seed yield and biomass production in crop and forage legumes.

## Introduction

Organ size is an important parameter in the characterisation of organ morphology and function. How plants control organ size is not only an intriguing, fundamental question in developmental biology, but it is also a crucial issue for improving grain yield and biomass production in crops. However, the genetic and molecular mechanisms that determine final organ or organism size are still poorly understood in plants.

Plant organ growth is mainly controlled by two coordinated developmental processes: cell division and cell expansion (Horiguchi *et al.*, 2006). Leaf development provides an excellent model system for analysing the coordination of these two important processes. During leaf development in eudicot plants, cells throughout the leaf primordia primarily undergo general proliferative cell division. Subsequently, a front of cell cycle arrest occurs in the apex‐to‐base direction. Immediately after the formation of this arrest front, there is a gradient of cell differentiation and expansion (Donnelly *et al.*, 1999; Kazama *et al.*, 2010; Andriankaja *et al.*, 2012). Although most cells begin to differentiate and enlarge, meristematic cells divide continuously to generate specific cell types within each cell layer, such as meristemoid and procambium cells, which are required for the formation of stomatal stem cells and vascular cells, respectively (White, 2006; Bergmann & Sack, 2007; Pillitteri & Torii, 2012). In *Arabidopsis thaliana*, meristemoid cells generate *c*. 67% of all pavement cells in cotyledons and 48% in leaves (Geisler *et al.*, 2000), implying that meristemoid cell division contributes significantly to final epidermal area in plants.

Molecular genetic studies in diverse plant species have revealed a number of key factors involved in regulating organ size by regulating cell division rate (Achard *et al.*, 2009; Rojas *et al.*, 2009; Eloy *et al.*, 2011; Zhang *et al.*, 2015), the duration of cell division (Hu *et al.*, 2003; Palatnik *et al.*, 2003; Kim & Kende, 2004; Horiguchi *et al.*, 2005; Disch *et al.*, 2006; Anastasiou *et al.*, 2007; Dewitte *et al.*, 2007; Li *et al.*, 2008; Rodriguez *et al.*, 2010; Li *et al.*, 2012; Xia *et al.*, 2013; Du *et al.*, 2014), or cell expansion (Kim *et al.*, 1998; Kim *et al.*, 1999; Hu *et al.*, 2006; Deprost *et al.*, 2007; Kurepa *et al.*, 2009; Sonoda *et al.*, 2009; Xu & Li, 2011; Lu *et al.*, 2014). Notably, recent studies on a series of organ and seed size mutants have revealed that the ubiquitin‐proteasome pathway plays an important role in plant organ size control. For example, the ubiquitin‐binding protein DA1 functions synergistically with the E3 ubiquitin ligases DA2 and BIG BROTHER (BB)/ENHANCER OF DA1 (EOD1) to control organ and seed size by limiting cell proliferation (Disch *et al.*, 2006; Li *et al.*, 2008; Xia *et al.*, 2013). UBIQUITIN‐SPECIFIC PROTEASE15 (UBP15), encoded by *SUPPRESSOR2 OF DA1* (*SOD2*), positively regulates seed size by promoting cell proliferation, and DA1 physically associates with UBP15 to modulate its stability (Du *et al.*, 2014). In *A. thaliana*, *STERILE APETALA* (*SAP*)/*SUPPRESSOR OF DA1* (*SOD3*) encodes an F‐box protein that functions as a component of the SKP1/Cullin/F‐box (SCF) E3 ubiquitin ligase complex (Wang *et al.*, 2016). SAP interacts with PPDs (PEAPOD1 and PEAPOD2) and KIXs (kinase‐inducible domain interacting proteins KIX8 and KIX9) and targets the KIX–PPD repressor complex for degradation to regulate organ growth (Gonzalez *et al.*, 2015; Wang *et al.*, 2016; Li *et al.*, 2018). Similarly, in *Capsella rubella*, decreasing SAP activity shortens the cell proliferation period and reduces the number of petal cells (Sicard *et al.*, 2016), while disrupting the cucumber (*Cucumis sativus* L.) *SAP* orthologue *LITTLELEAF* (*LL*) reduces leaf, flower, and fruit size and seed weight but increases lateral branch formation (Yang *et al.*, 2018), which is not observed in *A. thaliana sap*/*sod3* mutants, pointing to both the conserved and diverse roles of *SAP* in controlling lateral organ growth among plant species.

Legumes comprise one of the largest monophyletic families, with *c*. 700 genera and 18 000 species (Dong *et al.*, 2005). Legumes are second only to grasses in terms of economic and nutritional value (Graham & Vance, 2003). In addition to serving as important sources of protein and oils for the human diet, legumes are used as livestock forage and silage and as soil‐enhancing green manure (Graham & Vance, 2003) through fixing atmospheric nitrogen in association with rhizobial bacteria. However, due to an ancient genome duplication event, most important legume crops and forages, such as soybean (*Glycine max*) and alfalfa (*Medicago sativa*), have complex genomic structures (Shoemaker *et al.*, 2006). Thus, the genetic and molecular mechanisms that determine organ size in legumes are largely elusive.

A key regulator of seed size and seed weight, *BIG SEEDS1* (*BS1*), was recently identified in the diploid model legume plant *Medicago truncatula* (Ge *et al.*, 2016). *BS1* encodes a TIFY family transcriptional regulator related to tandemly repeated PPDs in *A. thaliana* that regulate the sizes of leaves and fruits but not seeds (White, 2006; Wang *et al.*, 2016). Notably, loss of function of *BS1* leads to enlarged seeds, as well as fruits and leaves, in *M. truncatula*, whereas the downregulation of *BS1* orthologues in soybean significantly increases seed size, weight and quality (Ge *et al.*, 2016), pointing to the great potential of using *BS1* for legume crop improvement. Despite this progress, the role of *BS1* in regulating organ size in legumes is still poorly understood.

In this study, we isolated and characterised the *M. truncatula* mutant *small leaf and bushy1* (*slb1*), which exhibits smaller leaves and petals but more lateral branches than the wild‐type. *SLB1* is an orthologue of *A. thaliana SAP*, encoding an F‐box protein that forms part of a SKP1/Cullin/F‐box E3 ubiquitin ligase complex. We demonstrate that SLB1 controls organ size and lateral branching by modulating the stability of BS1. Finally, overexpressing *SLB1* increased seed and leaf size in both *M. truncatula* and soybean, suggesting that *SLB1* represents a new genetic tool for increasing grain yield and biomass production in legume crops.

## Materials and Methods

### Plant materials and growth conditions

*Medicago truncatula* strain R108 and soybean cultivar Williams 82 were used for all experiments described in this study. *slb1‐1* (NF11180), *slb1‐2* (NF20634), and *slb1‐3* (NF19156) were identified from a *Tnt1* retrotransposon‐tagged mutant collection of *M. truncatula* R108. Plants were grown in a glasshouse under the following conditions: 24°C : 22°C, 16 h : 8 h, day : night photoperiod, and 60–70% relative humidity. The primers used to identify the *Tnt1* insertions are listed in Supporting Information Table S1.

### Morphological analysis

Projected area of leaves, petals and seeds were measured by scanning to generate digital images, followed by analysis using Olympus CellSens Standard 1.14 and imagej software (https://imagej.nih.gov/ij/). Cell numbers were calculated by dividing the leaf area by the average cell area.

### Vector construction and plant transformation

To generate the constructs used for complementation, a 7878‐bp fragment was amplified from *M. truncatula* R108 gDNA using the primers gSLB1‐F and gSLB1‐R and ligated to the pCAMBIA2300 vector (after digestion with *Eco*RI and *Sma*I) using the In‐Fusion cloning system (Clontech, Mountain View, CA, USA), yielding *gSLB1*; this construct contained the 2400‐bp upstream sequence, the entire gene sequence and the 650‐bp downstream sequence of *SLB1*. The 2400‐bp *SLB1* promoter was cloned into the pBGWFS7 vector using the Gateway system (Invitrogen) to generate the *pSLB1:GUS* (β‐glucuronidase) construct. CRISPR/Cas9 vector construction was performed as previously described (Meng *et al.*, 2017). Briefly, the *U6* promoter and single guide RNA (sgRNA) scaffold were amplified using the primer sets MtU6‐F1/MtU6‐R1 and BS1‐sgRNA‐F/R1, respectively. The *U6* promoter and sgRNA scaffold were integrated by PCR using the primers MtU6‐F1/R1 and ligated into the linearised destination vector pFGC5941 digested with *Xba*I using the In‐Fusion cloning system. The green fluorescent protein (*GFP*)*‐SLB1* fragment was amplified by fusing the coding sequence (CDS) of *GFP* with *SLB1* using the primers GFP‐attB1‐F and SLB1‐attB2‐R, followed by cloning into the binary vector pEarlygate203 using the Gateway system to generate the *35S:GFP‐SLB1* construct. The constructs were introduced into *Agrobacterium tumefaciens* by chemical transformation. *Agrobacterium tumefaciens* strain AGL1 was used for *M. truncatula* transformation, and strain EHA105 was used for soybean transformation as described (Tadege *et al.*, 2011; Chen *et al.*, 2018). All primers used are listed in Table S1.

### RNA extraction and gene expression analysis

Total RNA was extracted from various organs of *M. truncatula* plants using TRIzol Reagent (Invitrogen). cDNA was generated by reverse transcription with SuperScript (Invitrogen). Reverse transcription PCR (RT‐PCR) was performed using a 2× *Taq* PCR Master Mix (UPTECH) using *MtActin* as a control. Quantitative RT‐PCR was performed as previously described (Wang *et al.*, 2017) with at least three biological and three technical replicates for both the samples and controls. All primers used are listed in Table S1.

### Histochemical GUS staining

The GUS staining assay was performed as described (Niu *et al.*, 2015), and images of the GUS staining patterns of tissues were collected with a digital camera mounted on an Olympus SZX‐16 stereoscope.

### Subcellular localisation and confocal microscopy

To determine the subcellular localisation of SLB1, *A. tumefaciens* strain GV2260 containing *35S:GFP‐SLB1* and the nuclear marker plasmid *mRFP‐AHL22* were simultaneously infiltrated into 4‐wk‐old *Nicotiana benthamiana* leaves (Xiao *et al.*, 2009); *pMDC32‐GFP* was used as a control. P19 from tomato bushy stunt virus was used to inhibit transgene silencing. The fluorescence signals were observed 48–60 h after infiltration under a Zeiss LSM 700 confocal microscope.

### Sequence alignment and phylogenetic analysis

Sequence alignment was performed using ClustalW (http://www.genome.jp/tools/clustalw/). Bootstrap values of 1000 permutations for the neighbour‐joining phylogenetic tree were generated using mega 6.0 software (http://www.megasoftware.net/).

### Yeast two‐hybrid (Y2H) and bimolecular fluorescence complementation (BiFC) assays

The Y2H assay was performed using the Matchmaker Gold System (Clontech). The coding sequence of *SLB1* was cloned into the pGBKT7 vector, and the coding sequences of *MtASK1*, *MtASK2*, and *BS1* were cloned into the pGADT7 vector using the Gateway system (Invitrogen). The bait and prey plasmids were cotransformed into yeast strain Y2H Gold (Clontech). For the auxotrophic assay, yeast colonies were inoculated onto SD/−Leu/−Trp (DDO) and SD/−Trp/−Leu/−His/−Ade (QDO) plates and incubated in the dark at 28°C for 3 d. All primers used are listed in Table S1.

The BiFC assay was conducted as described (Meng *et al.*, 2019). Briefly, *SLB1* was cloned into pEarlygate201‐YN, while *MtASK1*, *MtASK2* and *BS1* were cloned to pEarlygate202‐YC using the Gateway system (Invitrogen). *SLB1‐nYFP*, *MtASK1‐cYFP*, *MtASK2‐cYFP* and *BS1‐cYFP* were introduced into *A. tumefaciens* strain GV2260. Various combinations of transformed *A. tumefaciens* cells were simultaneously infiltrated into 4‐wk‐old *N. benthamiana* leaves. P19 from tomato bushy stunt virus was used to inhibit transgene silencing. Fluorescence signals were observed 48–60 h after infiltration under a Zeiss LSM 700 confocal microscope.

### Co‐immunoprecipitation (Co‐IP) assay

A Co‐IP assay was performed as previously described with minor modifications (Meng *et al.*, 2019). The CDSs of *MtASK1*, *MtASK2*, *MtCUL1*, and *MtCUL2* were cloned into binary vector pGWB17 using the Gateway system (Invitrogen) to generate *35S:MtASK1‐Myc*, *35S:MtASK2‐Myc*, *35S:MtCUL1‐Myc*, and *35S:MtCUL2‐Myc*. *A. tumefaciens* strain GV2260 containing different combinations of *35S:GFP‐SLB1*, *35S:MtASK1‐Myc*, *35S:MtASK2‐Myc*, *35S:MtCUL1‐Myc*, *35S:MtCUL2‐Myc*, and *35S:GFP* constructs were infiltrated into *N. benthamiana* leaves. Total proteins were extracted from the samples with extraction buffer (50 mM Tris‐HCl, pH 7.5, 1 mM ethylene diamine tetraacetic acid (EDTA), 150 mM NaCl, 0.05–0.1% Tween 20, 10% glycerol, 1× protease inhibitor cocktail, and 1 mM phenylmethylsulfonyl fluoride (PMSF)). After protein extraction, 50 μl of anti‐GFP Magarose beads (Smart Life Sciences, Changzhou, Jiangsun, China) were added to the protein extract, and the mixture was incubated for 4 h at 4°C. The beads were washed at least four times with protein extraction buffer, and the proteins were eluted by adding SDS protein loading buffer and boiling the samples for 10 min. The clear supernatant was analysed by SDS‐PAGE and examined by immunoblot analysis using anti‐GFP (Abmart M20004S, 1/5000) and anti‐Myc (Abmart M20002L, 1/5000) antibodies.

### Degradation assay

For the degradation assay, the CDS of *BS1* and *MtWD40‐1* were cloned into binary vector pEarlygate202 and pGWB17 using the Gateway system to generate *35S:Flag‐BS1* and *35S:MtWD40‐1‐Myc*, respectively. *N. benthamiana* leaves transiently expressing *35S:Flag‐BS1* and *35S:GFP‐SLB1* or *35S:GFP* or *35S:MtWD40‐1‐Myc* were harvested and ground into a fine powder. Here, *c.* 1 g of *N. benthamiana* tissue was homogenised in 5 ml of native extraction buffer (50 mM Tris‐MES (2‐(N‐morpholino)ethanesulfonic acid), pH 8.0, 0.5 M sucrose, 1 mM MgCl_2_, 10 mM EDTA, 5 mM DTT, 10 mM ATP, 0.1% NP‐40, and 1× protease inhibitor cocktail) and filtered through a 70‐μm nylon filter. The lysates were incubated at 30°C, and samples were taken at the indicated time points. The samples were centrifuged at 6000 ***g*** for 1 min and the supernatant removed. Loading buffer was adding to the precipitate, followed by boiling for 10 min. For the proteasome inhibitor assay, MG132 (200 μM) or dimethyl sulfoxide (as a control) was added to the lysates. Protein levels were analysed by immunoblotting with anti‐FLAG antibody (MBL M185‐7, 1/5000).

### Pollen staining and *in vitro* pollen germination

Pollen staining was performed as previously described (Alexander, 1969). Flowers of wild‐type and *slb1‐1* were collected, fixed in Carnoy's fixative for 2 h, and stained with Alexander's solution for 2 h at room temperature. The samples were destained in 10% glycerol for 45 min before observation.

For pollen germination tests, drops of pollen germination medium (10% sucrose, 0.015% boric acid) were added on a microscope slide, pollens from the mature anthers of wild‐type and *slb1‐1* were sprinkled on the medium. The slide was placed on wet filter paper in moist plastic dish and incubated for 1.5 h at room temperature. The pollen tubes were imaged using an Olympus SZX‐16 stereoscope.

### GenBank accession numbers

GenBank accession numbers are as follows: MN954689 for SLB1 (Medtr5g097060), KM668032 for BS1 (Medtr1g102900), XP_003612225.1 for MtASK1 (Medtr5g022710), XP_003612227.1 for MtASK2 (Medtr5g022730), XP_013463300.1 for MtCUL1 (Medtr2g437390), XP_013458250.1 for MtCUL2 (Medtr4g119413), XP_003602392.1 for MtWD40‐1 (Medtr3g092840).

## Results

### Identification and characterisation of *M. truncatula slb1* mutants

**Figure 1:**
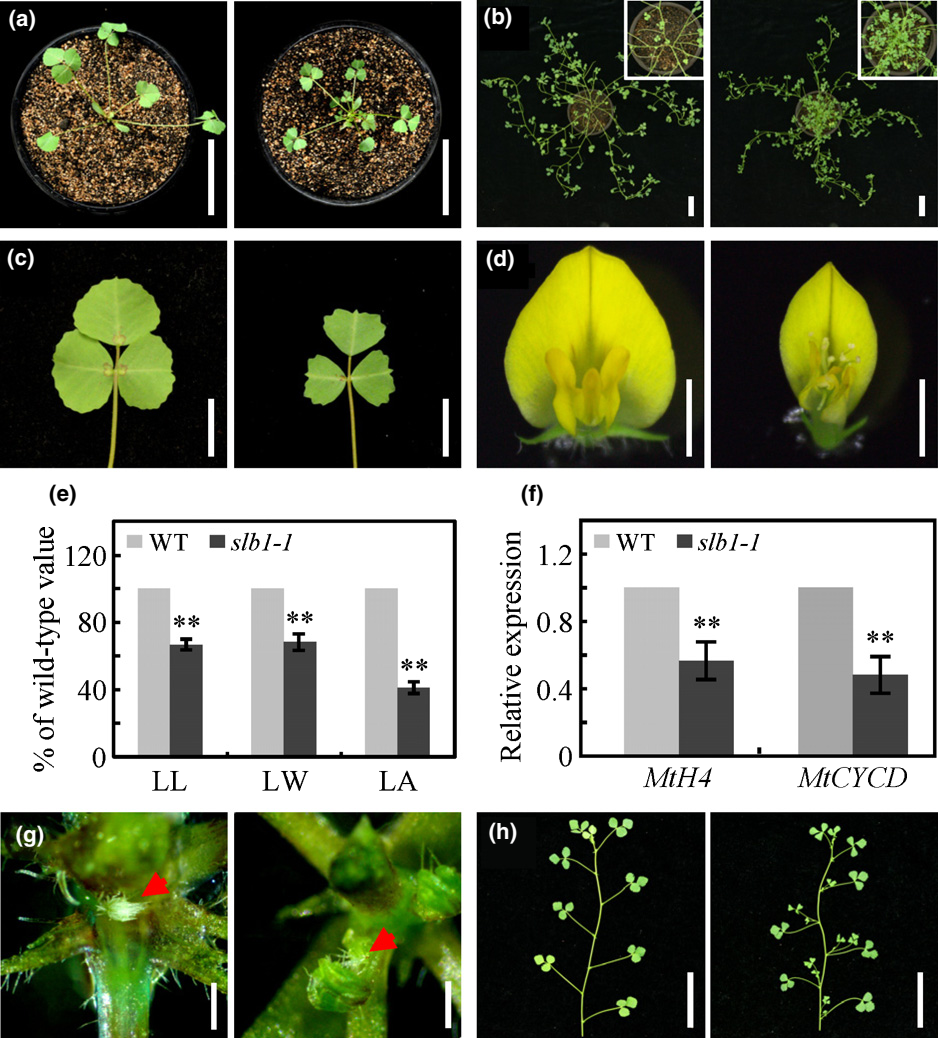
Morphological comparison of *Medicago truncatula* wild‐type vs *slb1‐1* plants. (a) 3‐wk‐old wild‐type (WT, left) and *slb1‐1* (right) seedlings. Bars, 5 cm. (b) 10‐wk‐old WT and *slb1‐1* plants. The insets show close‐up views of the shoot base. Bars, 5 cm. (c, d) Phenotypes of leaves (c) and flowers (d) in WT (left) and *slb1‐1* (right). Bars, 1 cm for leaves and 2 mm for flowers. (e) Leaf length (LL), leaf width (LW), and leaf area (LA) of WT and *slb1‐1*. Bars represent means ± SD (*n* = 16); asterisks indicate significant differences from the WT (**P < 0.01, Student's *t*‐test). (f) Transcript levels of *MtH4* and *MtCYCD* in *slb1‐1.* Bars represent means ± SD (*n* = 3); asterisks indicate significant differences from the WT (**P < 0.01, Student's *t*‐test). (g) Comparison of axillary bud formation between WT (left) and *slb1‐1* (right). Red arrows indicate axillary buds. Bars, 0.5 mm. (h) Dissected WT (left) and *slb1‐1* (right) branches, showing accelerated bud elongation in *slb1‐1*. Bars, 5 cm.

To investigate the molecular mechanisms underlying the control of organ size in legumes, we identified a mutant with obviously reduced leaf size named *small leaf and bushy1‐1* (*slb1‐1*) from a forward genetic screen of *Tnt1* retrotransposon‐tagged *M. truncatula* genotype R108 plants (Fig. 1a–c) (Tadege *et al.*, 2008; Yarce *et al.*, 2013). By contrast with the wild‐type, the *slb1‐1* mutant exhibits arrested leaf expansion in both the longitudinal and transverse directions, leading to reduced leaf size and heart‐shaped blade morphology (Fig. 1c,e). The *slb1‐1* mutant also exhibited smaller flowers compared with the wild‐type (Figs 1d, S1). A close examination showed that *slb1‐1* has a small carpel and retarded anthers, even though the dissected pollens could germinate normally, while the carpel of *slb1‐1* exhibited aberrant development with reduced and abnormal ovules, resulting in sterility (Fig. S2). Further examination of leaf epidermal cells in *slb1‐1* showed that the cell size was not obviously altered in this mutant, suggesting that its altered leaf size and shape are mainly caused by reduced cell proliferation (Fig. S3). Consistent with this notion, the transcript levels of the cell division markers *Histone H4* and *cyclin D* were significantly reduced in *slb1‐1* leaves, as determined by quantitative RT‐PCR (Fig. 1f). In addition, *slb1‐1* has more lateral branches than the wild‐type due to accelerated axillary bud outgrowth rather than increased axillary bud formation (Fig. 1a,b,g,h).

### Molecular cloning of the *SLB1* gene

**Figure 2:**
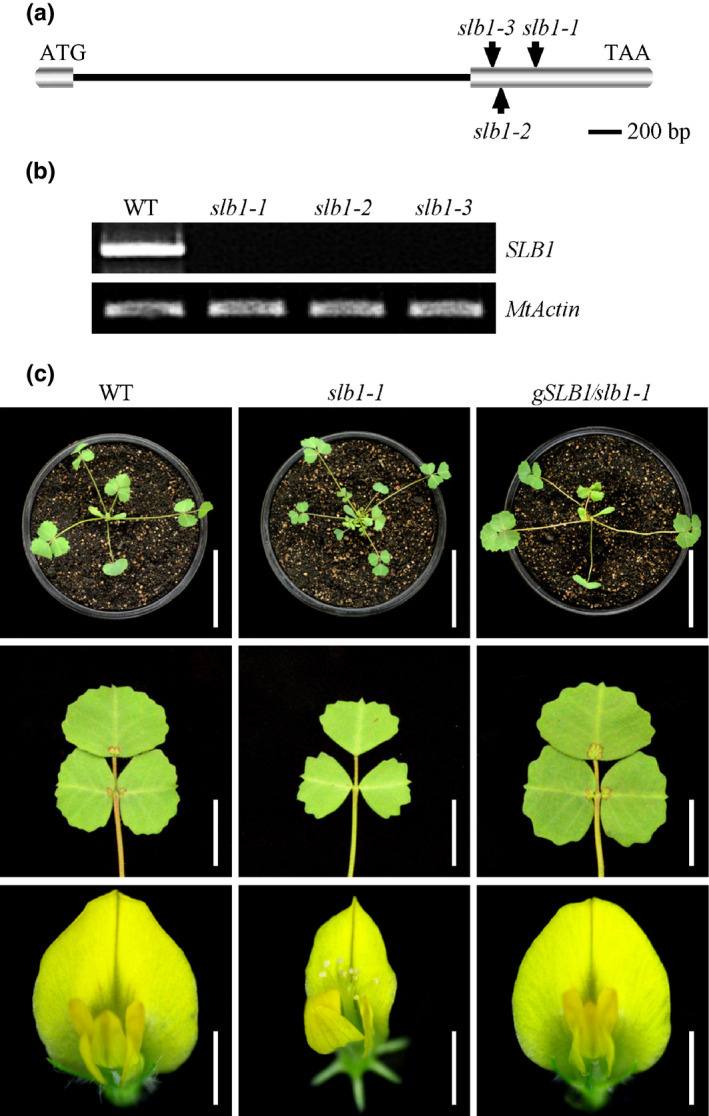
Cloning of the *Medicago truncatula SLB1* gene. (a) Schematic representation of the gene structure of *SLB1* and the *Tnt1* insertion sites in *slb1‐1*, *slb1‐2* and *slb1‐3*. (b) RT‐PCR analysis of *SLB1* expression in the wild‐type (WT) and various *slb1* alleles. *MtActin* was used as the loading control. (c) Phenotype analysis of branches (top panel), leaves (middle panel), and flowers (lower panel) of WT, *slb1‐1*, and *slb1‐1* plants complemented with *gSLB1* (*gSLB1/slb1‐1*). Bars, 5 cm in the top panel, 1 cm in the middle panel, and 2 mm in the lower panel.

The *slb1‐1* mutant was backcrossed with the wild‐type plants. All of the F1 progeny were wild‐type‐like, and in a segregating F_2_ population, the wild‐type‐like and mutant plants showed a segregation ratio of 3 : 1 (115 : 47), suggesting that the mutant phenotype was controlled by a single recessive gene. To identify the gene responsible for the pleiotropic defects in *slb1‐1*, the *Tnt1* flanking sequence tags (FSTs) of *slb1‐1* mutant were obtained from *Medicago truncatula* Mutant Database (https://medicago-mutant.noble.org/mutant/index.php) and analysed by PCR‐based genotyping in segregating populations (Tadege *et al.*, 2008). The FST, NF11180A_high_3, which segregated with the mutant phenotype of *slb1‐1*, was analysed by blast searches against the *M. truncatula* genome at NCBI (http://www.ncbi.nlm.nih.gov/) and Phytozome (https://phytozome.jgi.doe.gov/pz/portal.html) to obtain the full‐length sequence of *SLB1*. PCR and RT‐PCR were carried out to amplify the SLB1 genomic and coding sequences (CDS), respectively. The *SLB1* gene contains two exons, with the *Tnt1* retrotransposon inserted at the middle of exon 2 in *slb1‐1* 632 bp upstream of the translational stop codon (Fig. 2a). RT‐PCR revealed that the full‐length coding sequence of *SLB1* was not transcribed in *slb1‐1* (Fig. 2b). To confirm the notion that the *slb1‐1* mutant phenotype is caused by disruption of *SLB1*, we obtained two additional *Tnt1* insertion lines (NF20634 and NF19156) with the same phenotypes as described for *slb1‐1* via phenotypic screening (Fig. S4). Analysis of flanking sequences showed that NF20634 and NF19156 contained *Tnt1* insertions at different locations in exon 2 of *SLB1*; we therefore named these lines *slb1‐2* and *slb1‐3*, respectively (Fig. 2a). RT‐PCR analysis revealed that the transcripts of *SLB1* were abolished in these two mutants (Fig. 2b). The identity of *SLB1* was further confirmed by genetic complementation. We introduced a construct including the 2.4‐kb promoter region, the entire *SLB1* genomic DNA sequence, and the 0.65‐kb downstream region of *SLB1* (*gSLB1*) into *slb1‐1* plants by *A. tumefaciens*‐mediated transformation. The phenotypes of the complemented transgenic *slb1‐1* plants (*gSLB1/slb1‐1*) were comparable with those of the wild‐type (Fig. 2c). Collectively, these data confirmed the notion that disrupting *SLB1* function resulted in the altered organ size and increased branching of the *slb1* mutants.

### Expression analysis of *SLB1* and subcellular localisation of SLB1

**Figure 3:**
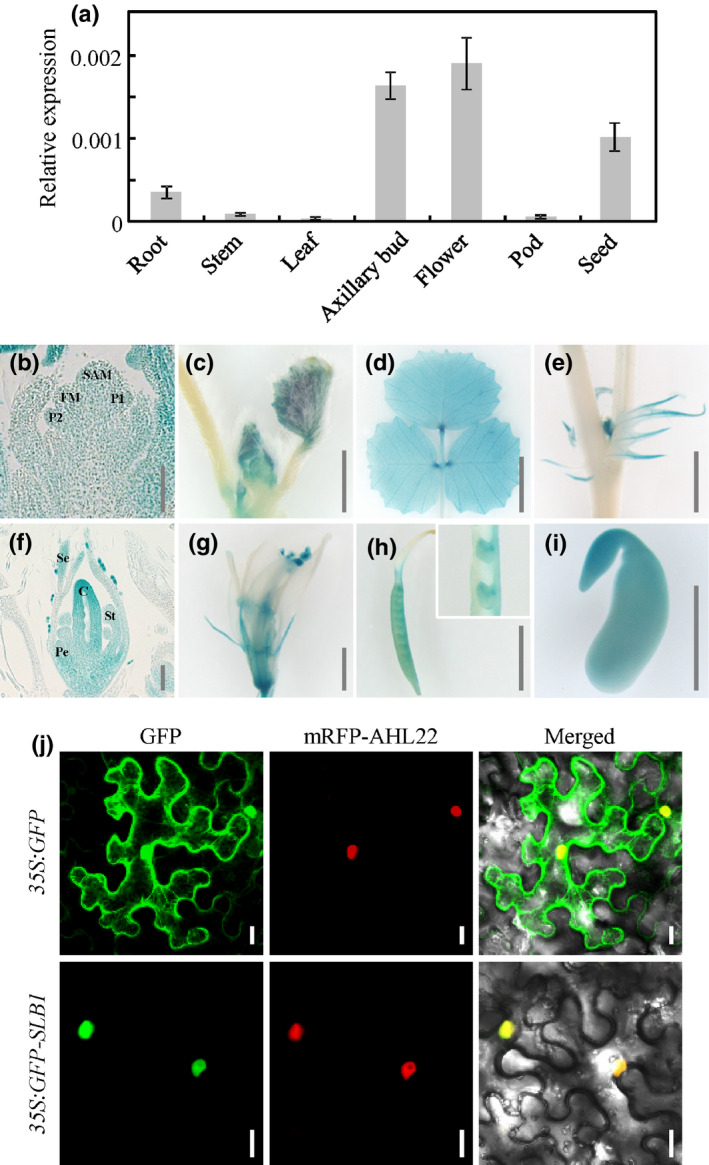
Expression pattern of *SLB1* in *Medicago truncatula* and subcellular localisation of SLB1 protein. (a) *SLB1* transcript levels in different tissues, as revealed by quantitative RT‐PCR. *MtActin* was used as an internal control. Bars represent means ± SD (*n* = 3). (b–i) GUS activity analysis in different tissues of wild‐type plants transformed with the *pSLB1:GUS* construct. GUS staining in shoot apical meristem (SAM), floral meristem (FM), and leaf primordia (P) (b), developing leaves (c, d), axillary bud (e), young floral bud (f), mature flower (g), carpel and dissected ovules (h), and immature seed (i). C, carpel; Pe, petal; Se, sepal; St, stamen. Bars, 100 μm in (b, f), 2 mm in (c, e, g–i), 5 mm in (d). (j) Subcellular localisation of GFP and GFP–SLB1 in tobacco leaf epidermal cells. The nuclear protein AHL22 was used as nuclear localisation marker. Bars, 20 μm.

*SLB1* was expressed at high levels in flowers and axillary buds, moderate levels in seeds and roots, and low levels in leaves, stems and fruits, as revealed by quantitative RT‐PCR (Fig. 3a). To gain a better spatial and temporal resolution, we analysed the expression of the GUS reporter gene fused to the 2.4‐kb *SLB1* promoter fragment in transgenic *M. truncatula* plants. In the shoot apex, we found that GUS staining was observed in the shoot apical meristem, the floral meristem and emerging leaf primordia (Fig. 3b). During leaf development, higher GUS activity and transcript abundance of *SLB1* were detected in younger leaves than older ones (Figs 3c,d, S5a). GUS staining was also observed at the pulvinus regions where leaflets were attached (Fig. 3d). Buds developing in the leaf axils showed high GUS staining, and GUS activity was observed in stipules (Fig. 3e). In very young flower buds, GUS staining was detected in most floral organs including sepals, petals, stamens, and carpels (Fig. 3f). As the flower develops further, GUS staining was observed at anthers, developing ovules as well as immature seeds (Fig. 3g–i). Further quantitative RT‐PCR analysis showed that *SLB1* was highly expressed during the early stages of floral buds, but the levels were reduced at the later stages (Fig. S5b). The expression pattern of *SLB1* supports its function in controlling leaf and flower size as well as lateral branching in *M. truncatula*.

To determine the subcellular localisation of SLB1, we fused the N terminus of SLB1 with GFP under the control of the cauliflower mosaic virus (CaMV) *35S* promoter and transformed the construct into tobacco (*Nicotiana benthamiana*) leaf epidermal cells by agro‐infiltration. By contrast with the GFP control, which was localised to both the cytoplasm and nuclei of epidermal cells, the GFP–SLB1 fusion protein was mainly localised to the nucleus (Fig. 3j).

### SLB1 functions within an SCF complex

**Figure 4:**
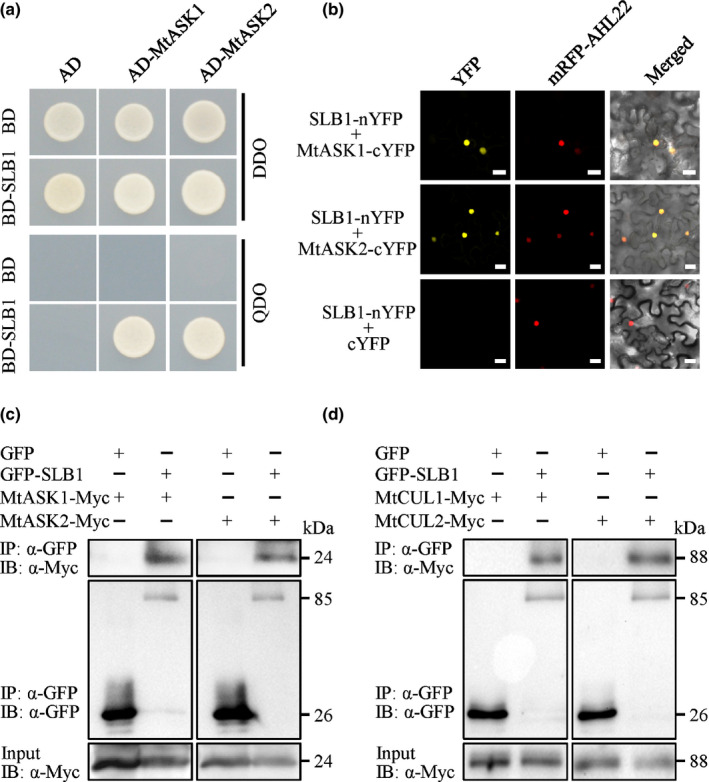
*Medicago truncatula* SLB1 physically associates with components of the SCF complex. (a) Interaction of SLB1 with MtASK1 and MtASK2 in an Y2H assay. Auxotrophic growth indicates the interaction of each protein. DDO and QDO indicate SD/−Trp/−Leu and SD/−Trp/−Leu/−His/−Ade, respectively. (b) Interaction of SLB1 with MtASK1 and MtASK2 in *Nicotiana benthamiana* leaf epidermal cells using a BiFC assay. AHL22 was used as a nuclear localisation marker. Bars, 20 μm. (c) SLB1 associates with MtASK1 and MtASK2 in *N. benthamiana*, as revealed in a Co‐IP assay. Total proteins were immunoprecipitated with anti‐GFP beads, and the immunoblots were probed with anti‐GFP and anti‐Myc antibodies. MtASK1‐Myc and MtASK2‐Myc were detected in the immunoprecipitated GFP–SLB1 complex. IP and IB indicate immunoprecipitation and immunoblot, respectively. (d) SLB1 associates with MtCUL1 and MtCUL2 in *N. benthamiana*, as revealed in a Co‐IP assay. Total proteins were immunoprecipitated with anti‐GFP beads, and the immunoblots were probed with anti‐GFP and anti‐Myc antibodies. MtCUL1‐Myc and MtCUL2‐Myc were detected in the immunoprecipitated GFP–SLB1 complex. IP and IB indicate immunoprecipitation and immunoblot, respectively.

Blast searches against the NCBI database showed that *SLB1* encodes an F‐box protein homologous to *A. thaliana* SAP. SLB1 and SAP share 43.7% amino acid sequence identity (using full‐length sequences), with strong conservation at the F‐box motif (Fig. S6). F‐box proteins have been shown to function as the substrate‐recruiting subunits of SKP1/Cullin/F‐box‐protein (SCF) complexes (Kipreos & Pagano, 2000; Cardozo & Pagano, 2004). Given that SLB1 contains the highly conserved F‐box motif, we asked whether SLB1 functions within an SCF complex in *M. truncatula*. We searched for potential SKP1 and Cullin‐related proteins in the *Medicago truncatula* Genome Database (http://www.medicagogenome.org) using the *A. thaliana* ASK1/2 and Cullin1 proteins as query sequences. We identified two SKP1‐like proteins (which we named MtASK1 and MtASK2) and two Cullin1‐like proteins (MtCUL1 and MtCUL2) (Fig. S7). SLB1 interacted with both MtASK1 and MtASK2 in an Y2H assay (Fig. 4a). These interactions were verified via a BiFC assay in *N. benthamiana* leaves using split YFP. Strong yellow fluorescence was clearly observed between SLB1 fused to the N‐terminal half of YFP (SLB1–nYFP) and MtASK1 or MtASK2 fused to the C‐terminal half of YFP (MtASK1–cYFP or MtASK2–cYFP), whereas no YFP fluorescent signals were detected in the negative control (combination of SLB1–nYFP and cYFP alone; Fig. 4b).

To explore whether SLB1 physically associates with an SCF complex *in planta*, we performed Co‐IP analysis to detect the interactions of SLB1 with MtASK1, MtASK2, MtCUL1 and MtCUL2 *in vivo*. We transiently co‐expressed *35S:GFP‐SLB1* with *35S:MtASK1‐Myc* or *35S:MtASK2‐Myc* in *N. benthamiana* leaves. Transient coexpression of *35S:GFP* with *35S:MtASK1‐Myc* or *35S:MtASK2‐Myc* was used as a negative control. We isolated total proteins from the samples, incubated them with anti‐GFP Magarose beads to immunoprecipitate GFP‐SLB1 and GFP, and used anti‐GFP and anti‐Myc antibodies to detect the immunoprecipitated proteins. MtASK1‐Myc and MtASK2‐Myc were captured in the immunoprecipitation complex containing GFP–SLB1, but not in the control, indicating that SLB1 physically associates with MtASK1 and MtASK2 *in planta* (Fig. 4c). When we transiently co‐expressed *35S:GFP‐SLB1* with *35S:MtCUL1‐Myc* or *35S:MtCUL2‐Myc* in *N. benthamiana* leaves, MtCUL1‐Myc and MtCUL2‐Myc were also detected in the immunoprecipitated GFP–SLB1 complex (Fig. 4d). These results suggested that SLB1 functions within an SCF ubiquitin ligase complex in *M. truncatula*.

### SLB1 physically interacts with BS1 and targets it for degradation

**Figure 5:**
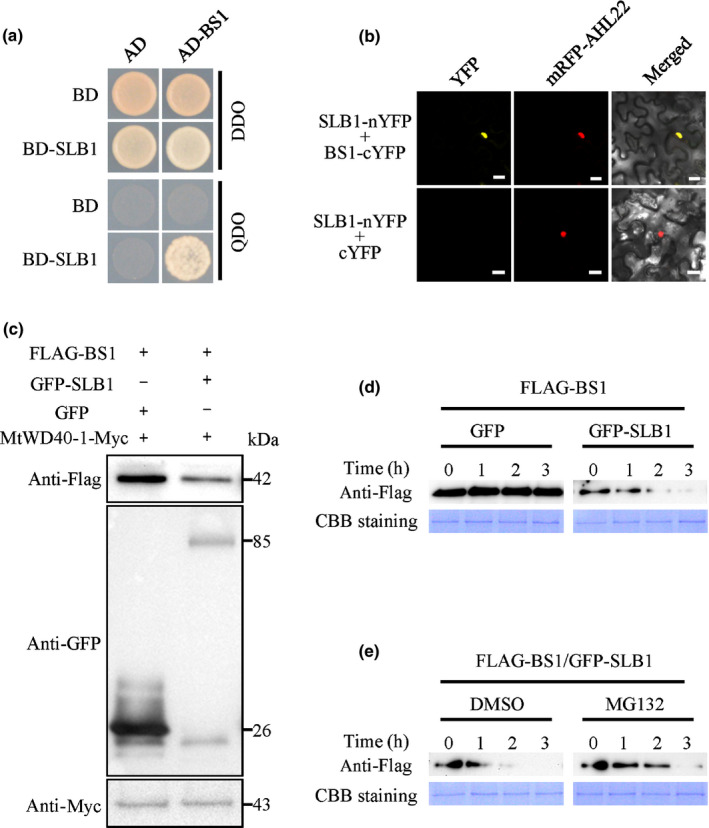
*Medicago truncatula* SLB1 interacts with and targets BS1 for degradation. (a) Interaction between SLB1 and BS1 in an Y2H assay. Auxotrophic growth indicates the interaction of each protein. DDO and QDO indicate SD/−Trp/−Leu and SD/−Trp/−Leu/−His/−Ade, respectively. (b) Interaction between SLB1 and BS1 in *Nicotiana benthamiana* leaf epidermal cells using a BiFC assay. AHL22 was used as a nuclear localisation marker. Bars, 20 μm. (c) SLB1 promotes the degradation of BS1 *in vivo*. Immunoblotting analysis of total protein corresponding to agro‐infiltrated *N. benthamiana* leaves with the indicated plasmids. The abundance of FLAG−BS1 was detected using anti‐FLAG antibody, and that of GFP−SLB1 was detected using anti‐GFP antibody. MtWD40‐1‐Myc detection using anti‐Myc antibody served as a loading control. (d) SLB1 promotes the degradation of BS1 in an *in vitro* protein degradation assay. Protein samples from tobacco leaves coexpressing FLAG–BS1 and GFP–SLB1 or GFP were incubated at 30°C for the indicated times. The abundance of FLAG–BS1 was detected using anti‐FLAG antibody. Coomassie Brilliant Blue (CBB) staining served as a loading control. (e) BS1 degradation is inhibited by MG132. Protein samples from tobacco leaves coexpressing FLAG–BS1 and GFP–SLB1 were treated with MG132 or dimethyl sulfoxide (DMSO, as control) at 30°C for the indicated times. The accumulation of FLAG‐BS1 protein was detected by immunoblotting with anti‐FLAG antibody. CBB staining served as a loading control.

SAP interacts with PPD proteins in *A. thaliana* and targets them for degradation to control organ size. Given that *BS1* is a *PPD* orthologue in *M. truncatula* that plays a conserved role in controlling organ size in legumes, we asked whether SLB1 physically interacts with BS1 and targets it for degradation. Expression pattern analyses showed that *BS1* transcripts were present in most tissues, including roots, stems, leaves, fruits and seeds and were highly expressed in flowers and axillary buds (Fig. S8a). Furthermore, the GFP–BS1 fusion protein primarily localised to the nucleus (Fig. S8b), indicating that both spatial expression and localisation of *BS1* are associated with that of *SLB1.* Furthermore, the Y2H assay showed that SLB1 interacts with BS1 (Fig. 5a) and this interaction was verified in *N. benthamiana* leaves via a BiFC assay using split YFP (Fig. 5b).

To investigate whether SLB1 modulates the stability of BS1 protein, we transiently expressed FLAG‐BS1 with GFP‐SLB1 or GFP in *N. benthamiana* leaves. Compared with coexpression with GFP, the stability of BS1 was reduced by coexpression with SLB1 (Fig. 5c). *In vitro* degradation experiments indicated that SLB1 promotes the degradation of BS1, which was suppressed by treatment with MG132, a 26S proteasome‐specific inhibitor (Fig. 5d,e). These results indicated that SLB1 promotes the degradation of BS1 via the ubiquitin‐26S proteasome‐dependent pathway.

### *SLB1* genetically interacts with *BS1* to control organ size and lateral branching

**Figure 6:**
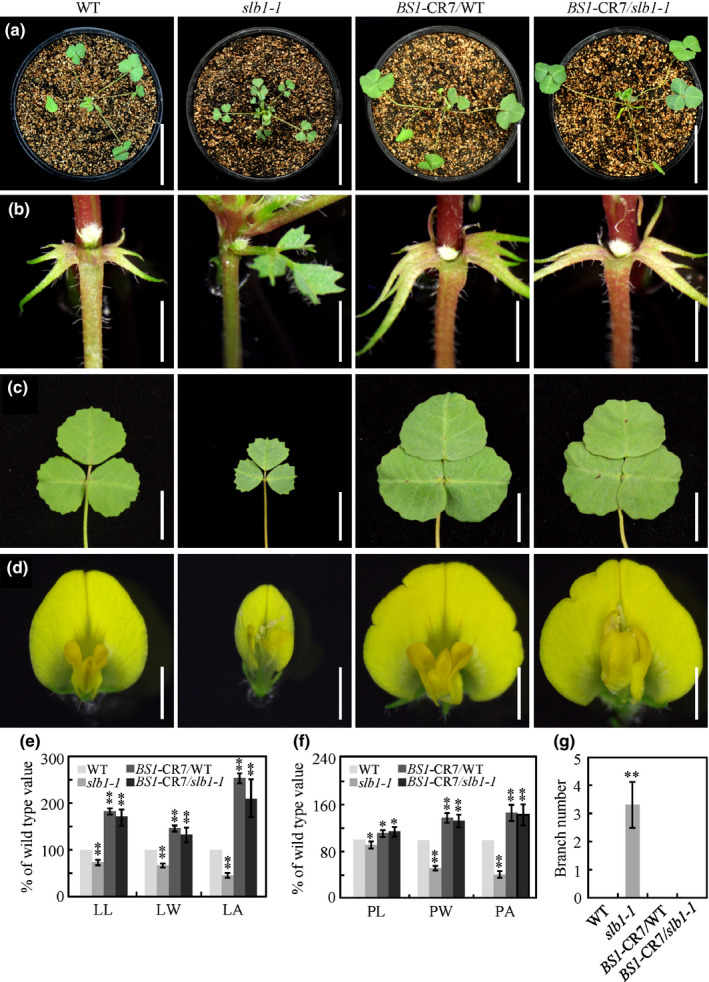
SLB1 genetically interacts with BS1 to control *Medicago truncatula* organ size and lateral branching. (a–d) Phenotypic analyses of branches (a), axillary buds (b), leaves (c), and flowers (d) in the wild‐type (WT), *slb1‐1*, *BS1‐*CR7/WT and *BS1‐*CR7/*slb1‐1*. Bars, 5 cm in (a), 2 mm in (b), 1 cm in (c) and 2 mm in (d). (e) Leaf length (LL), leaf width (LW), leaf area (LA) of WT, *slb1‐1*, *BS1‐*CR7/WT and *BS1‐*CR7/*slb1‐1* plants. Bars represent means ± SD (*n* = 15); asterisks indicate significant differences from the WT (**P < 0.01, Dunnett's test). (f) Vexillum petal length (PL), vexillum petal width (PW), vexillum petal area (PA) of WT, *slb1‐1*, *BS1‐*CR7/WT and *BS1‐*CR7/*slb1‐1* plants. Bars represent means ± SD (*n* = 15); asterisks indicate significant differences from the WT (*P < 0.05, **P < 0.01, Dunnett's test). (g) Branch number of 3‐wk‐old WT, *slb1‐1*, *BS1‐*CR7/WT and *BS1‐*CR7/*slb1‐1* seedlings. Branch indicates axillary bud length >5 mm. Bars represent means ± SD (*n* = 10); asterisks indicate significant differences from the WT (**P < 0.01, Dunnett's test).

As SLB1 associates with BS1 and regulates its stability, we investigated whether BS1 functions with SLB1 in a common pathway to control organ size and lateral branching in *M. truncatula*. Compared with the wild‐type, plants with disrupted expression of *BS1* in the R108 background, as generated by CRISPR/Cas9 (*BS1*‐CR), displayed an arrested axillary bud outgrowth, but exhibited obviously increased leaf and flower size (Figs 6a–g, S9), which is consistent with the phenotype of the previously reported *bs1‐1* mutant in the cv Jemalong A17 background (Ge *et al.*, 2016). By contrast, disrupting *BS1* in the *slb1* mutant background not only suppressed the reduced leaf and flower size phenotype, and the branching phenotype of *slb1‐1*, but also significantly increased the size of the leaves and flowers compared with wild‐type R108, which is similar to the phenotype of the *bs1* single mutant (Fig. 6a–g). These results clearly indicated that *BS1* is epistatic to *SLB1* with respect to the control of organ size and lateral branching in *M. truncatula.*

### Overexpression of *SLB1* results in increased leaf and seed size in *M. truncatula* and soybean

**Figure 7:**
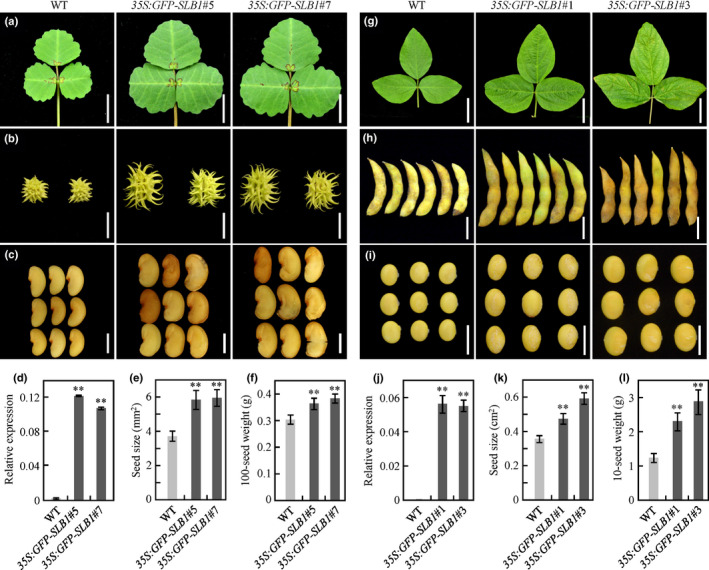
*SLB1* overexpression increases leaf, fruit and seed size in *Medicago truncatula* and soybean. (a–c) Phenotypes of leaves (a), fruits (b), and seeds (c) of wild‐type (WT) and *SLB1‐*overexpressing *M. truncatula*. Bars, 1 cm in (a) and (b) and 2 mm in (c). (d) Transcript levels of *SLB1* in transgenic *M. truncatula* plants, as revealed by quantitative RT‐PCR. *MtActin* was used as an internal control. Bars represent means ± SD (*n* = 3); asterisks indicate significant differences from the WT (**P < 0.01, Dunnett's test). (e) Comparison of seed size in WT and *SLB1*‐overexpressing *M. truncatula* plants. Bars represent means ± SD (*n* = 15); asterisks indicate significant differences from the WT (**P < 0.01, Dunnett's test). (f) Comparison of 100‐seed weight of WT and *SLB1*‐overexpressing *M. truncatula* plants. Bars represent means ± SD (*n* = 3); asterisks indicate significant differences from the WT (**P < 0.01, Dunnett's test). (g–i) Phenotypes of leaves (g), fruits (h), and seeds (i) of WT and *SLB1*‐overexpressing soybean. Bars, 5 cm in (g), 2 cm in (h), and 1 cm in (i). (j) Transcript levels of *SLB1* in transgenic soybean plants, as revealed by quantitative RT‐PCR. *GmActin11* was used as an internal control. Bars represent means ± SD (*n* = 3); asterisks indicate significant differences from the WT (**P < 0.01, Dunnett's test). (k) Comparison of seed size in WT and *SLB1*‐overexpressing soybean. Bars represent means ± SD (*n* = 15); asterisks indicate significant differences from the WT (**P < 0.01, Dunnett's test). (l) Comparison of 10‐seed weight of WT and *SLB1*‐overexpressing soybean. Bars represent means ± SD (*n* = 5); asterisks indicate significant differences from the WT (**P < 0.01, Dunnett's test).

Seed size is a major agronomic trait of crop plants. Given that *SLB1* and *BS1* function in a common genetic pathway, we wondered whether *SLB1* could be used to modify seed size in legume crops. To test this idea, we introduced the full‐length *SLB1* CDS into *M. truncatula* as well as the major legume crop soybean, under the control of the *35S* promoter via *A. tumefaciens*‐mediated transformation. Both the *SLB1‐*overexpressing transgenic *M. truncatula* and soybean plants showed significantly increased organ size, including leaves, fruits and seeds, compared with those of wild‐type plants (Fig. 7a–e,g–k). To quantitatively measure these improvements, we evaluated total above ground biomass yield of 9‐wk‐old wild‐type and *SLB1* overexpressing *M. truncatula* plants. The average fresh weight of *35S:GFP‐SLB1*#5 and *35S:GFP‐SLB1*#7 transgenic *M. truncatula* had a 1.35‐fold and 1.32‐fold increase, respectively, in total biomass compared with the wild‐type, while the total amount of dry biomass yield of *35S:GFP‐SLB1*#5 and *35S:GFP‐SLB1*#7 had increased by over 1.43‐fold and 1.37‐fold per plant, respectively (Fig. S10). Moreover, we evaluated the seed size and seed weight of *SLB1‐*overexpressing *M. truncatula* and soybean plants after maturity. The average seed size and seed weight of the *SLB1*‐overexpressing *M. truncatula* plants increased by 1.58‐fold and 1.22‐fold, respectively, compared with the wild‐type (Fig. 7e,f), while the seed size and seed weight increased by over 1.49‐fold and 2.08‐fold, respectively, in *SLB1*‐overexpressing transgenic soybean (Fig. 7k,l). These results indicated that *SLB1* plays a conserved role in controlling leaf and seed size in these plants, suggesting that it could be used to increase grain and biomass yield in legume crops.

## Discussion

### Functional conservation and divergence of *M. truncatula SLB1* and *A. thaliana SAP* in controlling organ growth

In the model eudicot *A. thaliana*, SAP was initially identified as a floral regulator, as the loss‐of‐function *sap* mutant exhibited severe defects in inflorescence, flower and ovule development, leading to sterile flowers with reduced petal size (Byzova *et al.*, 1999). Another allelic mutant of *SAP*, *sod3‐1*, showed reduced leaf, flower and fruit size due to decreased cell numbers, indicating that SAP also functions as a master regulator of organ size (Wang *et al.*, 2016). Similarly, in this study, we demonstrated that disrupting *SLB1* in *M. truncatula* leads to reduced leaf and flower size as well as poor fertility with abnormal ovules (Figs 1c–e, S1, S2), suggesting that *SLB1* and *SAP* share a conserved role in regulating vegetative and reproductive organ development. Nevertheless, the *slb1* mutant produces more branches than the wild‐type (Fig. 1a,b), which was not observed in *A. thaliana sap* or *sod3* mutants. A genetic complementation experiment confirmed that this additional branching phenotype is caused by the disruption of *SLB1* function (Fig. 2c), indicating that *SLB1* is also involved in regulating branch development in *M. truncatula.* Notably, recent studies in cucumber showed that disrupting *Leaf length *(*LL*), an orthologue of *SAP*, leads to small organ size but the formation of multiple lateral branches (Yang *et al.*, 2018), suggesting that *SLB1* and *LL* have pleiotropic effects on branching in both *M. truncatula* and cucumber, although the regulation of organ size appears to be a conserved function of *SAP/LL/SLB1* in diverse plant species. These results are in agreement with previous phylogenetic analysis showing that SLB1 and LL group together, but distinct from the closely related SAP (Yang *et al.*, 2018), implying a functional divergence of SAP orthologues in *M. truncatula* and cucumber.

SAP controls organ growth in *A. thaliana* by targeting PPD and KIX for degradation (Wang *et al.*, 2016; Li *et al.*, 2018). Our results indicated that SLB1 can function within an SCF complex (Fig. 4) and physically interact with BS1, a PPD homologue in *M. truncatula*, and modulate its stability (Fig. 5), suggesting that SLB1 uses a repressor module similar to PPD‐KIX‐TPL to control organ size. Supporting this scenario, disrupting *BS1* using CRISPR/Cas9 suppressed the leaf and flower phenotypes of *slb1* (Figs 6, S9). However, by contrast with the *ppd* mutant, which only partially suppressed the organ growth phenotypes of *A. thaliana sod3‐1*, disrupting *BS1* in the *slb1* mutant background not only recovered the organ growth phenotype but also resulted in enlarged leaves and flowers (Fig. 6c–f). These findings suggested that *SLB1* controls organ size in *M. truncatula* primarily by modulating the stability of BS1. Given the different regulatory modules present in *M. truncatula* vs *A. thaliana*, the identification and characterisation of *M. truncatula KIX* genes should shed light on the roles of the SAP‐PPD‐KIX regulatory module in diverse plant systems.

### SLB1 controls *M. truncatula* lateral branching by modulating the stability of BS1

F‐box proteins play a variety of roles in regulating phytohormone signalling and stress responses during plant development. For example, the F‐box proteins TRANSPORT INHIBITOR RESPONSE1 (TIR1) and CORONATINE‐INSENSITIVE PROTEIN1 (COI1) are involved in auxin and jasmonic acid signalling, respectively (Dharmasiri *et al.*, 2005; Kepinski & Leyser, 2005; Sheard *et al.*, 2010). Studies of a series of branching mutants, including *more axillary growth* (*max*) in Arabidopsis, *ramosus* (*rms*) mutants in pea, and *dwarf* (*d*) mutants in rice have revealed that the F‐box proteins MAX2/RMS4/D3 are responsible for perceiving and transducing strigolactone (SL) signals to regulate shoot branching (Stirnberg *et al.*, 2002; Ishikawa *et al.*, 2005; Johnson *et al.*, 2006; Stirnberg *et al.*, 2007). In this study, we demonstrated that the F‐box protein SLB1 acts as a novel regulator of lateral branching by modulating the stability of BS1 in *M. truncatula*. BS1 was previously shown to control organ size in *M. truncatula*, including seed, fruit and leaf size, via a regulatory module that targets primary cell proliferation (Ge *et al.*, 2016). The disruption of BS1 fully restored the branching phenotype of the *slb1* mutant (Fig. 6b,g), suggesting that BS1 has an epistatic effect on lateral branching. Thus, our findings define a novel genetic and molecular mechanism of the F‐box protein SLB1 and the organ size regulator BS1 in controlling lateral branching in *M. truncatula*.

The development of shoot branching generally comprises two distinct steps: the formation of the axillary meristems in the leaf axils and the outgrowth of axillary buds (Shimizu‐Sato & Mori, 2001). In some plant species, the outgrowth of axillary buds can be suppressed by the primary shoot, a phenomenon known as apical dominance (Sachs & Thimann, 1964; Cline, 1991). The phytohormones auxin and cytokinin have long been implicated in the process, in which auxin inhibited the outgrowth of axillary buds by affecting the supply of cytokinin to axillary buds (Eklof *et al.*, 2000; Li & Bangerth, 2003; Nordstrom *et al.*, 2004; Tanaka *et al.*, 2006). Notably, many flowering plants with sterility issues exhibited increased branching, which is thought to be an indirect effect of the auxin export levels from growing meristems vs fruits (Hensel *et al.*, 1994; Ware *et al.*, 2019). The observation of sterility and increased branching in the *slb1* mutant suggests that the *SLB1* regulation of lateral bud outgrowth may be connected to auxin transport. Moreover, recent studies with branching mutants in several plant species have demonstrated that SLs, a group of terpenoid lactones, are newly identified phytohormone that repress shoot branching (Gomez‐Roldan *et al.*, 2008; Umehara *et al.*, 2008). The *slb1* mutant showed defects in axillary bud dormancy, leading to accelerated lateral branch growth, while disrupting *BS1* restores the axillary bud dormancy of *slb1*, indicating that the SLB1‐BS1 regulatory module involves in the regulation of *M. truncatula* apical dominance, probably via affecting phytohormone crosstalk network.

It is worth noting that the *slb1* mutant shows a decreased cell proliferation in most lateral organs while an accelerated lateral bud outgrowth, suggesting the cell proliferation might be enhanced in lateral buds by involving additional regulators epistatic to *SLB1*. The TCP (TEOSINTE BRANCHED1/CYCLOIDEA/PCF) family genes may be interesting candidates for investigation for possible roles in SLB1–BS1‐mediated control of shoot branching in *M. truncatula*. For example, the *BRANCHED1* (*BRC1*) gene is thought to integrate various environmental and hormonal signals, including auxin, cytokinin and SL signalling, to regulate bud growth (Aguilar‐Martinez *et al.*, 2007; Braun *et al.*, 2012; Dun *et al.*, 2012; Barbier *et al.*, 2019). Further elucidating the actions of the SLB1–BS1 regulatory module and investigating their interactions with phytohormones, including auxin, cytokinin and SLs and other branching regulators, like *BRC1*, will enlighten our understanding of the genetic network underlying this fundamental process.

### Increasing seed yield by manipulating *SLB1* expression in legume crops

Organ size is an important agronomic trait that influences crop yields (Li *et al.*, 2019). Soybean, a major crop worldwide, provides up to 69% of proteins and 30% of oils in the human diet (Lam *et al.*, 2010; Zhou *et al.*, 2015). To meet the needs of the rapidly increasing human population, soybean breeders are faced with the challenge of designing a high‐efficiency breeding strategy for developing soybean varieties with higher yields and improved quality (Masuda & Goldsmith, 2009; Ray *et al.*, 2013). In fact, several laboratories are currently focused on increasing seed size via genetic engineering to improve soybean yields. Downregulating the *BS1* orthologues, *GmBS1* and *GmBS2*, using artificial microRNA was successfully used to modify seed size, thereby increasing soybean yields (Ge *et al.*, 2016). However, this type of knockout strategy is often limited due to the redundancy of gene targets, especially in legumes with complex genomes. As overexpressing *SLB1* resulted in enlarged seed and leaf phenotypes similar to those produced by downregulating *BS1* orthologues (Fig. 7), *SLB1* represents an alternative target for genetic manipulation of seed size, perhaps serving as a new tool for significantly improving seed yield in soybean and other legume crops.

## Author contributions

PY, LN and HL conceived and designed the research. PY, QM, HW and DF performed the experiments. XW, YP and JW contributed analytical tools. PY, MT, LN and HL analysed the data and wrote the manuscript. LN and HL contributed equally to this work. This work has not been submitted elsewhere for publication, and all authors reviewed the manuscript.

## Supporting information


**Fig. S1** Comparison of vexillum petal size of *M. truncatula* wild‐type and *slb1‐1*.
**Fig. S2** The *M. truncatula slb1* mutant shows defects in floral organ development.
**Fig. S3** Disruption of *SLB1* leads to significant decreases in leaf size, primarily by affecting cell proliferation in *M. truncatula*.
**Fig. S4** Phenotypes of the *M. truncatula* wild‐type and *slb1* alleles.
**Fig. S5** Transcript abundance of *SLB1* in *M. truncatula* leaves and flowers at different developmental stages.
**Fig. S6** Sequence alignment of *M. truncatula* SLB1 and Arabidopsis SAP.
**Fig. S7** Phylogenetic analysis of ASK1/2‐like and CUL1‐like family proteins in *M. truncatula*.
**Fig. S8** Expression pattern of *BS1* in *M. truncatula* and subcellular localisation of BS1.
**Fig. S9** Targeted mutagenesis of *M. truncatula BS1* using the CRISPR/Cas9 system.
**Fig. S10** *SLB1* overexpression improves biomass yield in *M. truncatula*.
**Table S1** Primers used in this study.
Please note: Wiley Blackwell are not responsible for the content or functionality of any Supporting Information supplied by the authors. Any queries (other than missing material) should be directed to the *New Phytologist* Central Office.
