# Supplementary material for: SMALL LEAF AND BUSHY1 controls organ size and lateral branching by modulating the stability of BIG SEEDS1 in Medicago truncatula
Source: New Phytol. 2020 Feb 19;226(5):1399–412. doi: 10.1111/nph.16449 (PMC7317789; doi:10.1111/nph.16449)
Supplement: Supplementary file 1 — Fig. S1 Comparison of vexillum petal size of M. truncatula wild‐type and slb1‐1. Fig. S2 The M. truncatula slb1 mutant shows defects in floral organ development. Fig. S3 Disruption of SLB1 leads to significant decreases in leaf size, primarily by affecting cell proliferation in M. truncatula. Fig. S4 Phenotypes of the M. truncatula wild‐type and slb1 alleles. Fig. S5 Transcript abundance of SLB1 in M. truncatula leaves and flowers at different developmental stages. Fig. S6 Sequence alignment of M. truncatula SLB1 and Arabidopsis SAP. Fig. S7 Phylogenetic analysis of ASK1/2‐like and CUL1‐like family proteins in M. truncatula. Fig. S8 Expression pattern of BS1 in M. truncatula and subcellular localisation of BS1. Fig. S9 Targeted mutagenesis of M. truncatula BS1 using the CRISPR/Cas9 system. Fig. S10 SLB1 overexpression improves biomass yield in M. truncatula. Table S1 Primers used in this study. Please note: Wiley Blackwell are not responsible for the content or functionality of any Supporting Information supplied by the authors. Any queries (other than missing material) should be directed to the New Phytologist Central Office. [file NPH-226-1399-s001.pdf]

New Phytologist Supporting Information Figs S1–S10 and Table S1

Article title: SMALL LEAF AND BUSHY1 controls organ size and lateral branching by modulating the stability of BIG SEED1 in *Medicago truncatula*

Authors: Pengcheng Yin, Qingxia Ma, Hui Wang, Dan Feng, Xianbing Wang, Yanxi Pei, Jiangqi Wen, Million Tadege, Lifang Niu and Hao Lin

Article acceptance date: 11 January 2020

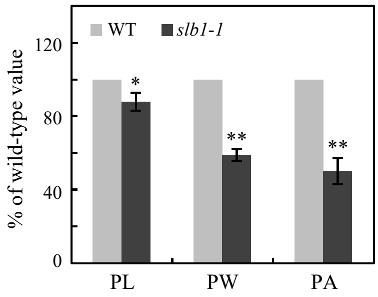

Fig. S1 Comparsion of vexillum petal size of *M. truncatula* wild type and *slb1-1*. Vexillum petal length (PL), vexillum petal width (PW), vexillum petal area (PA) of the wild type (WT) and *slb1-1*. Bars represent means ± SD (n = 15); asterisks indicate significant differences from WT (*$P < 0.05$, **$P < 0.01$, Student's t test).

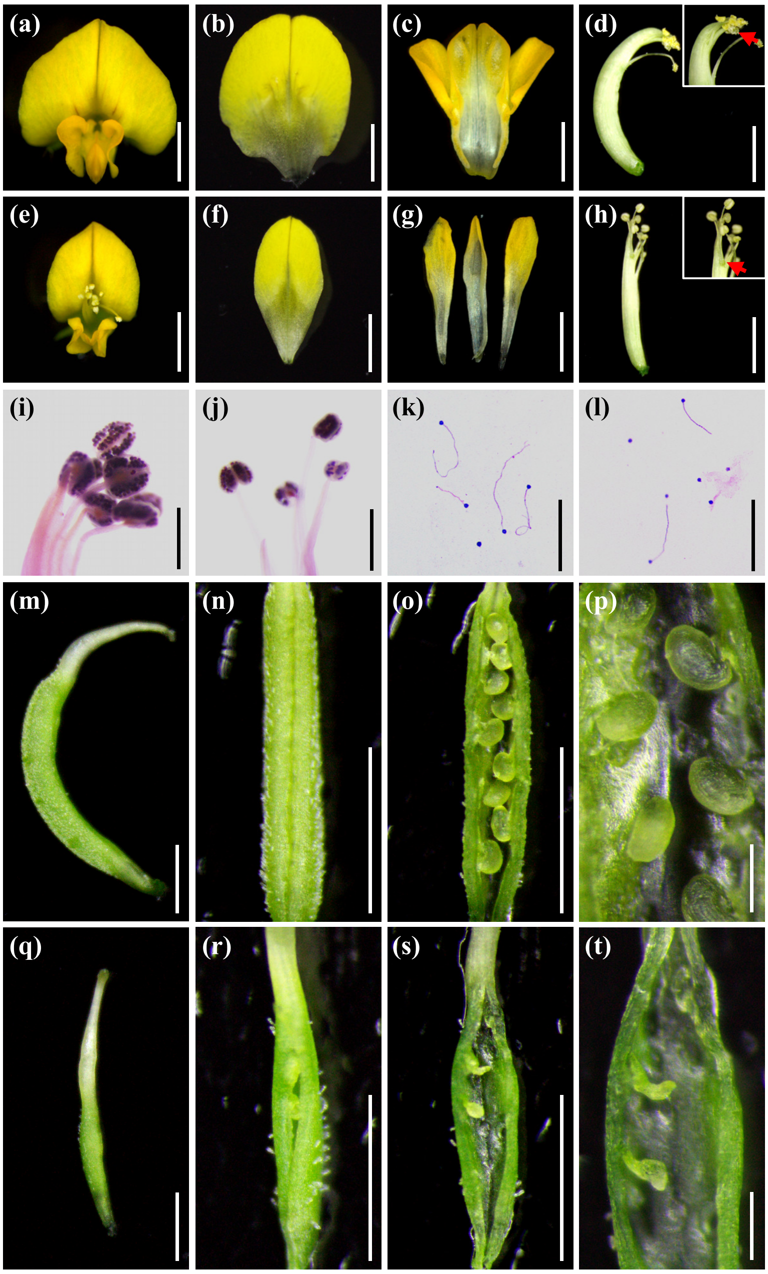

Fig. S2 The *M. truncatula slb1* mutant shows defects in floral organ development. (a-d) The mature flower (a), dissected vexillum petal (b), the fused alae and keel petals (c), and the stamens and carpel (d) of wild-type (WT) *M. truncatula*. The inset is the magnification of anthers. Red arrow indicates the stigma. Bars = 2 mm. (e-h) The mature flower (e), dissected vexillum petal (f), the alae and keel petals (g), and the stamens and carpel (h) of *slb1-1*. The inset is the magnification of anthers. Red arrow indicates the stigma. Bars = 2 mm. (i, j) Pollen staining in WT (i) and *slb1-1* (j). Bars = 0.5 mm. (k, l) *In vitro* pollen tube germination of WT (k) and *slb1-1* (l). Bars = 0.5 mm. (m-p) The carpels (m, n) and ovules (o, p) of WT. Bars = 1 mm in (m-o), and 0.2 mm in (p). (q-t) The carpels (q, r) and ovules (s, t) of the *slb1-1* mutant. Bars = 1 mm in (q-s), and 0.2 mm in (t).

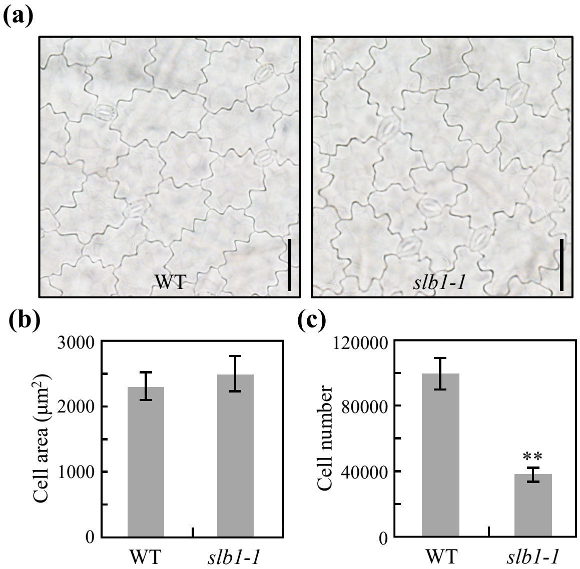

Fig. S3 Disruption of *SLB1* leads to significant decreases in leaf size, primarily by affecting cell proliferation in *M. truncatula*.

(a) Micrographs of cleared wild-type (WT) and *slb1-1* leaves. Bars = 50 μm. (b) Comparison of cell area in WT and *slb1-1* leaves. Bars represent means ± SD (n = 15). (c) Comparison of cell number in WT and *slb1-1* leaves. Bars represent means ± SD (n = 15); asterisks indicate significant differences from the WT (**P < 0.01, Student's t test).

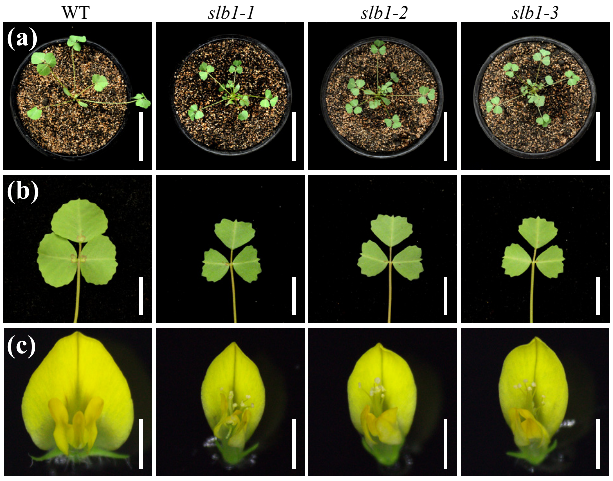

Fig. S4 Phenotypes of the *M. truncatula* wild type and *slb1* alleles.
(a) 3-week-old seedlings of the wild type (WT) and *slb1* alleles. Bars = 5 cm. (b, c) Phenotypes of leaves (b) and flowers (c) of WT and *slb1* mutants. Bars = 1 cm for leaves and 2 mm for flowers.

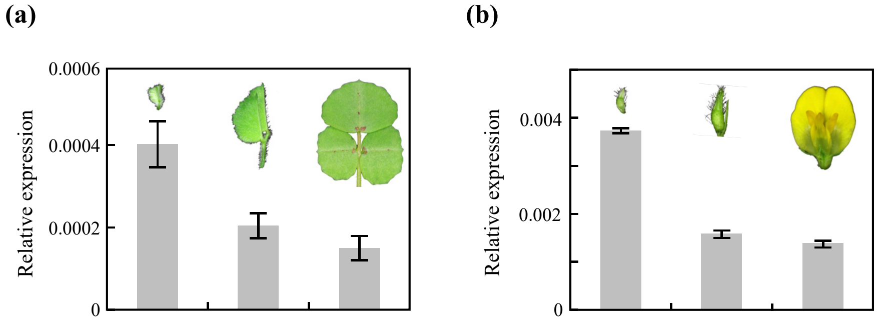

Fig. S5 Transcript abundance of *SLB1* in *M. truncatula* leaves and flowers at different developmental stages.

Quantitative RT-PCR analysis of *SLB1* expression in leaves (a) and flowers (b) at different developmental stages. Bars represent means ± SD (n = 3).

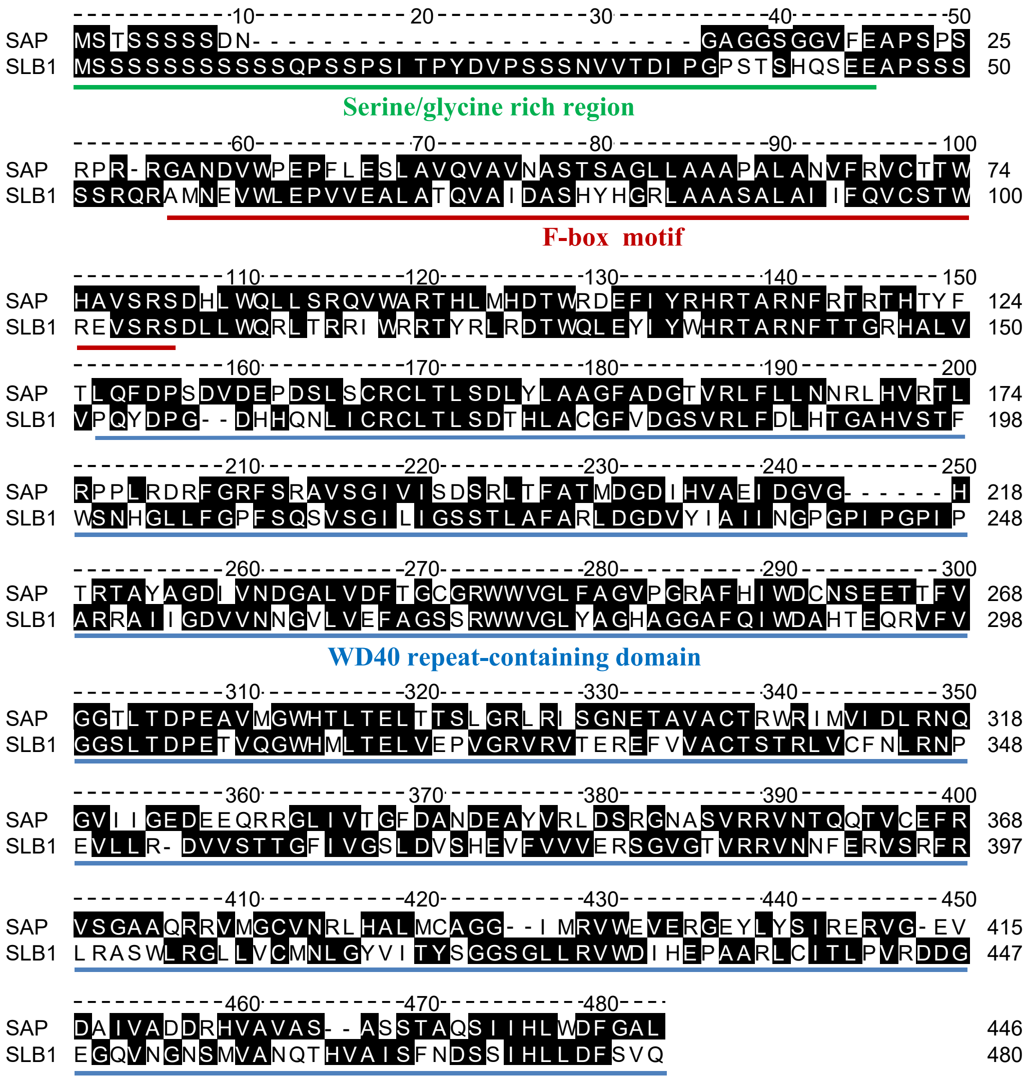

Fig. S6 Sequence alignment of *M. truncatula* SLB1 and Arabidopsis SAP.
The conserved serine/glycine rich region, F-box motif, and WD40 repeat-containing domain sequences are underlined in green, red, and blue, respectively.

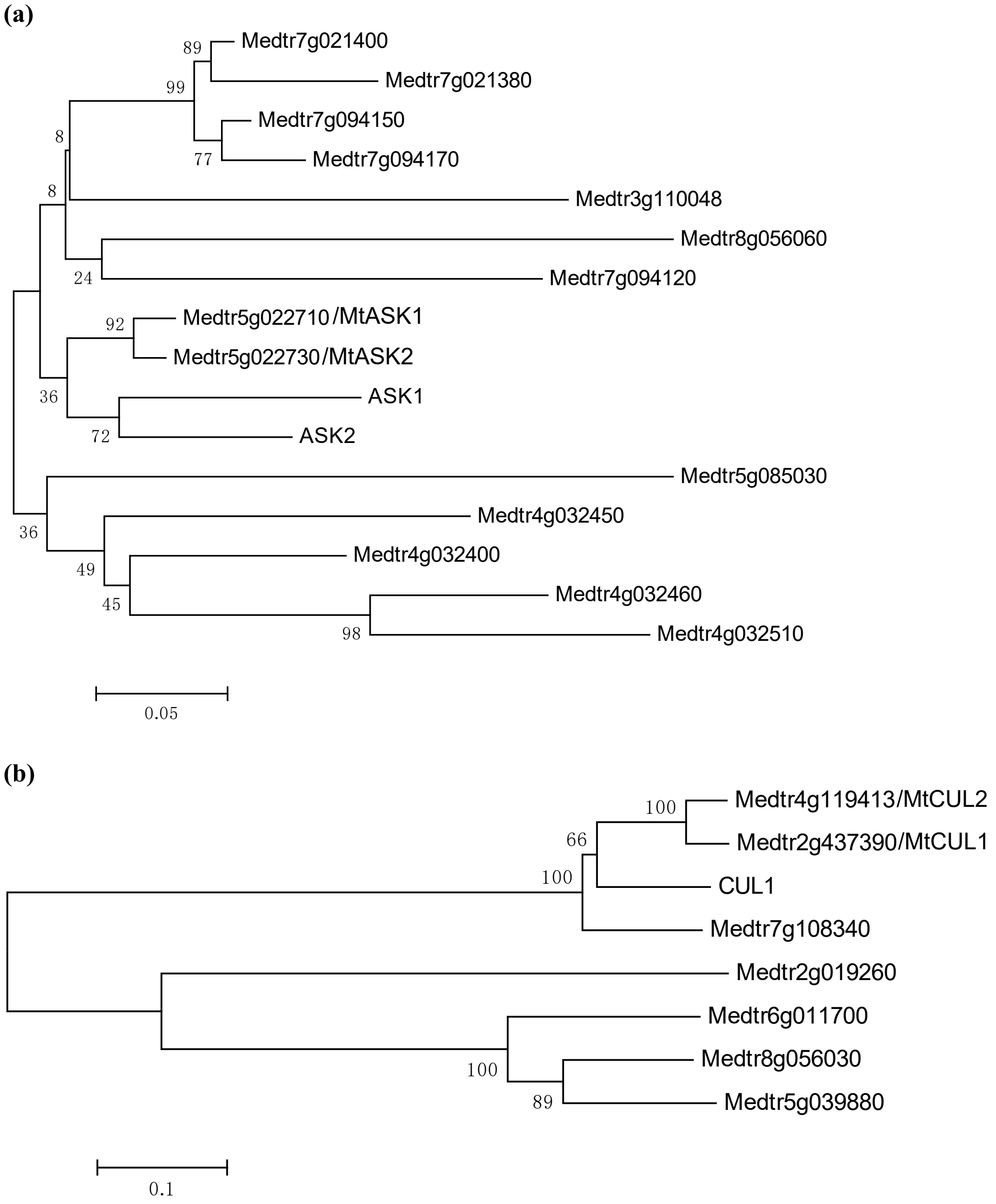

Fig. S7 Phylogenetic analysis of ASK1/2-like and CUL1-like family proteins in *M. truncatula.*

Phylogenetic analysis of *M. truncatula* ASK1/2-like (a) and CUL1-like (b) proteins. The protein sequences were obtained from the *Medicago truncatula* Genome Database (http://www.medicagogenome.org). Full-length amino acid sequences were aligned using ClustalW, and the trees were constructed using MEGA6.0 with 1000 replicates to generate bootstrap values.

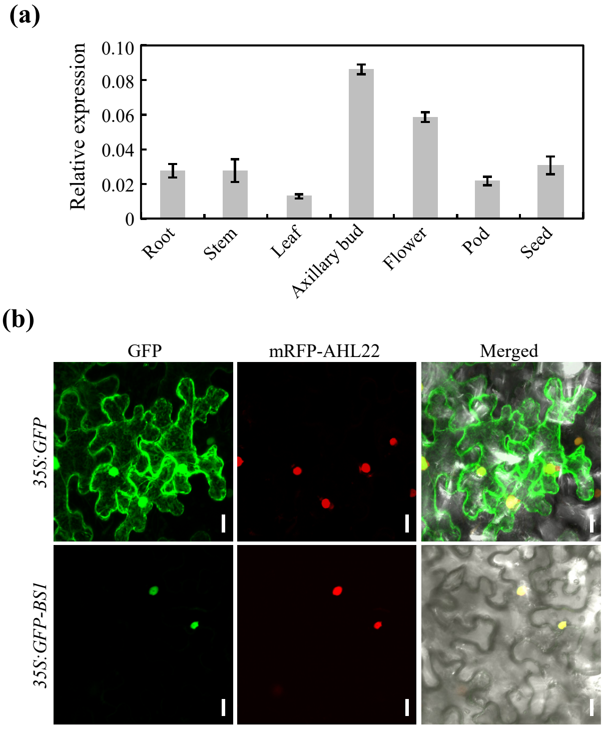

Fig. S8 Expression pattern of *BS1* in *M. truncatula* and subcellular localization of BS1. (a) Transcript levels of *BS1* in different *M. truncatula* tissues, as revealed by quantitative RT-PCR. *MtActin* was used as an internal control. Bars represent means ± SD (*n* = 3). (b) Subcellular localization of GFP and GFP-BS1 in tobacco leaf epidermal cells. The nuclear protein AHL22 was used as a nuclear localization marker. Bars = 20 μm.

(a)

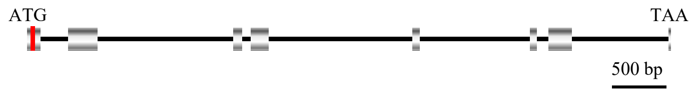

(b)

WT (R108):	GAGTTGGTTAAGAGGTCTGTCGAGGATG--GATCGGAAGGAAACGGTG	
BS1-CR4/WT:	GAGTTGGTTAAGAGGTCTGTCGAGGATGCGATCGGAAGGAAACGGTG	+1
BS1-CR4/WT:	GAGTTGGTTAAGAGGTCTGTCGAGGATG----ATCGGAAGGAAACGGTG	-1
BS1-CR7/WT:	GAGTTGGTTAAGAGGTCTGTCGAGGATG----ATCGGAAGGAAACGGTG	-1
BS1-CR7/WT:	GAGTTGGTTAAGAGGTCTGTCGAGGATG----ATCGGAAGGAAACGGTG	-1
BS1-CR1/*slb1-1*:	GAGTTGGTTAAGAGGTCTGTCGAGGATG----ATCGGAAGGAAACGGTG	-1
BS1-CR1/*slb1-1*:	GAGTTGGTTAAGAGG--------------------------GATCGGAAGGAAACGGTG	-13
BS1-CR2/*slb1-1*:	GAGTTGGTTAAGAGGTCTGTCGAGGATGTGATCGGAAGGAAACGGTG	+1
BS1-CR2/*slb1-1*:	GAG--- AAACGGTG	-35
BS1-CR3/*slb1-1*:	GAGTTGGTTAAGAGGTCTGTCGAGGATG----ATCGGAAGGAAACGGTG	-1
BS1-CR3/*slb1-1*:	GAGTTGGTTAAGAGGTCTGTCGAGGA--------------------------AACGGTG	-13
BS1-CR7/*slb1-1*:	GAGTTGGTTAAGAGGTCTGTCGAGGATG----ATCGGAAGGAAACGGTG	-1
BS1-CR7/*slb1-1*:	GAGTTGGTTAAGAGGTCTGTCGAGGATG----ATCGGAAGGAAACGGTG	-1

Fig. S9 Targeted mutagenesis of *M. truncatula BS1* using the CRISPR/Cas9 system.
(a) Schematic representation of the gene structure of *BS1*. The red line indicates the CRISPR/Cas9 target site. (b) Nucleotide sequence of wild-type (WT) *BS1* aligned to the sequences of the mutant alleles. Red font indicates base deletions or insertions; the protospacer adjacent motifs are underlined.

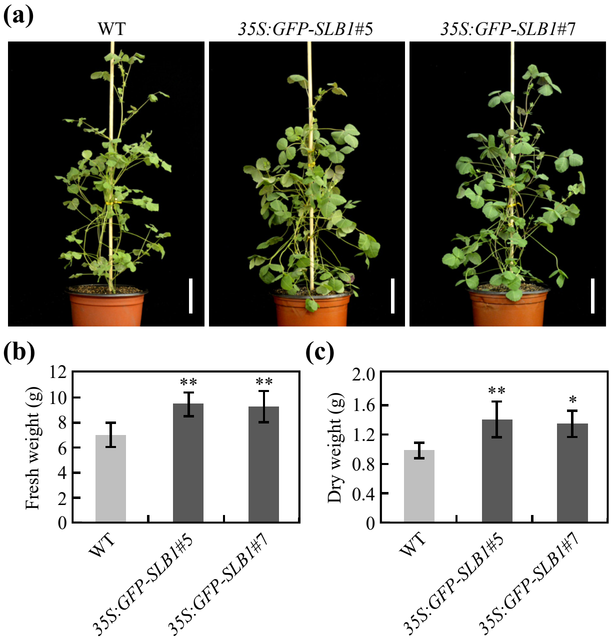

Fig. S10 *SLB1* overexpression improves biomass yield in *M. truncatula*.
(a) Morphology of 9-week-old wild-type (WT) and *SLB1* overexpressing *M. truncatula* plants. Bars = 5 cm. (b, c) Comparison of fresh (b) and dry (c) weights of aboveground biomass in 9-week-old WT and *SLB1* overexpressors. Bars represent means ± SD (n = 5); asterisks indicate significant differences from WT (*P < 0.05, **P < 0.01, Dunnett's test).

Table S1. Primers used in this study

Primers	Sequences 5'-3'	Application
SLB1-NF11180/NF19156-F	ATGGTTGGAAAATGCATGC	For genotyping of *slbl-1* and *slb1-3*
SLB1-NF11180/NF19156-R	ATTATTCACGCGCCGAACA	
SLB1-NF20634-F	TTTGTTCAACATGGAGGGAA	For genotyping of *slb1-2*
SLB1-NF20634-R	AGTTCCAACACCACTTCTTT	
LTR6	GCTACCAACCAAACCAAGTCAA	Primers in *Tnt1* for genotyping of different mutant
LTR31	CTCCTCTCGGGGTCGTGGTT	
gSLB1-F	CCATGATTACGAATTCCAACCGTCAACATGAAATGAATCCA	To amplify gDNA of *SLB1*
gSLB1-R	GTCGACAGATCCCCGGGACTAATATGTGGTCTTGCTAGCCA	
pSLB1-F	CACCCAACCGTCAACATGAAATGAATCCA	To amplify promoter of *SLB1*
pSLB1-R	GAGGAGTGAGAGAAGTGGAGGG	
GFP-attB1-F	GGGGACAAGTTTGTACAAAAAAGCAGGCTTCATGGGTAAAGGAGAACTTTTCA	To amplify CDS of *GFP*
6*Gly-GFP-R	GCCACCCCCTCCGCCACCGGCATAATCAGGCACATCG	
6*Gly-SLB1-F	GGTGGCGGAGGGGGTGGCATGTCTTCTTCTTCCTCCTCCT	To amplify CDS of *SLB1*
SLB1-attB2-R	GGGGACCACTTTGTACAAGAAAGCTGGGTCTTACTGTACACTAAAATCCAATA	
BS1CDS-F	CACCATGAACGGCGGAAGCACCGTTTCCT	To amplify CDS of *BS1*
BS1CDS-R	TTAGCATTCTTGAACATCTTTATCATTC	
MtU6-F1	GCTTAGGCCTTCTAGAATCCAACATTTCACTTGAGTTAACT	To amplify *MtU6* promoter
MtU6-R1	AAACCCTGCTGTTCGTCTAG	
BS1-sgRNA-F	CTAGACGAACAGCAGGGTTTGAGGTCTGTCGAGGATGGATGTTTTAGAGCTAGAAATAG	To amplify sg RNA-scaffold fragment
R1	GGCAACGCGTTCTAGAAAAAAAAGCACCGACTCGGTGC	
6*Gly-BS1-F	GGTGGCGGAGGGGGTGGCATGAACGGCGGAAGCACCGTTTCCT	To amplify CDS of *BS1*
BS1-attB2-R	GGGGACCACTTTGTACAAGAAAGCTGGGTCTTAGCATTCTTGAACATCTTTATCA	
MtASK1-5'UTR-F	AGGGTTAGCGATTTCACAAA	To amplify CDS and UTR of *MtASK1*
MtASK1-3'UTR-R	TCTACATTGCGGAACTTTGA	

MtASK2-5'UTR-F	TCGAAAAGGGGAAGAACAAT	To amplify CDS and UTR of *MtASK2*
MtASK2-3'UTR-R	AGATCCTTGACTTAGATGCT	
MtASK-F	CACCATGTCTTCAACAAGAAAGATCACTCT	To amplify CDS of *MtASK1* and *MtASK2*
MtASK-R	TTCAAATGCCCATTGGTTTTCCCTAC	
MtCUL1-F	CACCATGAGTGAACGGAAGACTATTG	To amplify CDS of *MtCUL1*
MtCUL1-R	AGCCAAGTACTTGAACAAATTTGCATTA	
MtCUL2-F	CACCATGTCAATGAGTGAAAGGAAAA	To amplify CDS of *MtCUL2*
MtCUL2-R	AGCTAAGTACTTGAACATATTTGGATTCTC	
MtWD40-1-attB1-F	GGGGACAAGTTTGTACAAAAAAGCAGGCT TCATGGATAATTCAACACAAGAATCCC	To amplify CDS of *MtWD40-1*
MtWD40-1-attB2-R	GGGGACCACTTTGTACAAGAAAGCTGGGT CAACCCTCAAAAGCTGCATT	
RT-SLB1-F	CACCATGTCTTCTTCTTCCTCCTCCT	For RT-PCR analysis of *SLB1*
RT-SLB1-R	CTGTACACTAAAATCCAATA	
RT-MtActin-F	TCTTACTCTCAAGTACCCCATTGAGC	For RT-PCR analysis of *MtActin*
RT-MtActin-R	GTGGGAGTGCATAACCCTCATAGATT	
qSLB1-F	GGCCACTCAAGTTGCCATTG	For quantitative RT-PCR analysis of *SLB1*
qSLB1-R	ATCCGAACGTGACACTTCCC	
qMtCYCD-F	TCTTGGATGGAAGATGAGTCCAGC	For quantitative RT-PCR analysis of *MtCYCD*
qMtCYCD-R	ACATGAACCATTGTAGCAGTTGCC	
qMtH4-F	AAGGGTGGTGCTAAGCGTCACCGC	For quantitative RT-PCR analysis of *MtH4*
qMtH4-R	TGTTCAGTGTAAGTGACAGCATCACG	
qRT-MtActin-F	TCAATGTGCCTGCCATGTATGT	For quantitative RT-PCR analysis of *MtActin*
qRT-MtActin-R	ACTCACACCGTCACCAGAATCC	
qGmActin11-F	ATCTTGACTGAGCGTGGTTATTCC	For quantitative RT-PCR analysis of *GmActin11*
qGmActin11-R	GCTGGTCCTGGCTGTCTCC	
